# Sustainable Concrete Production Using Granodiorite, Alkali Feldspar Granite, and Mafic Metavolcanic Rock Powders as Supplementary Cementitious Materials

**DOI:** 10.3390/ma19112193

**Published:** 2026-05-22

**Authors:** A. Serag Faried, Nourhan Fathy, W. M. Morsi, Maher Dawoud, Abdelhalim S. Mahmoud, Khaled M. Osman

**Affiliations:** 1 Civil Engineering Department, Faculty of Engineering, Fayoum University, Fayoum 63514, Egypt; nf1293@fayoum.edu.eg (N.F.); kma04@fayoum.edu.eg (K.M.O.); 2 Department of Physics, College of Science, Imam Mohammad Ibn Saud Islamic University (IMSIU), Riyadh 11432, Saudi Arabia; wammohamed@imamu.edu.sa; 3 Geology Department, Faculty of Science, Menofia University, Shebin El Koum 32511, Egypt; dawoud_99@yahoo.com; 4 Geology Department, Faculty of Science, Fayoum University, Fayoum 63514, Egypt; asm07@fayoum.edu.eg

**Keywords:** granodiorite, alkali feldspar granite, mafic metavolcanic, supplementary cementitious materials, mechanical properties, microstructure

## Abstract

This study aims to explore the effect of using three distinct silicate- and aluminate-rich rock powders—granodiorite (GDP), alkali-feldspar granite (AFGP), and mafic metavolcanic (MMVP)—sourced from Egypt’s largely unexploited Eastern Desert geological resources, as supplementary cementitious materials (SCMs) in concrete production. Rock samples were processed into ultrafine powders (1.4–1.5 μm average particle size) and utilized as partial cement replacements at 3%, 6%, 9%, and 12% by weight. These rock powders were confirmed to meet ASTM C618 requirements for natural pozzolans, qualifying them as viable SCMs. Pozzolanic activity was confirmed through Strength Activity Index (SAI) testing, with values of 79%, 82%, and 76% for GDP, AFGP, and MMVP, respectively, all exceeding the 75% minimum threshold required by ASTM C618. Fresh concrete workability decreased progressively with increasing rock powder content. Mechanical testing demonstrated optimal replacement levels of 9% for GDP and AFGP, and 6% for MMVP, achieving 28-day compressive strength improvements of 14.1%, 16.0%, and 14.9%, respectively, compared to plain Portland cement concrete without any rock powder replacement (control mix). Splitting tensile strength increased by 14.7%, 12.7%, and 16.3% at optimal dosages. Microstructural analysis via SEM revealed enhanced matrix densification and reduced porosity through physical filler effects and pozzolanic reactions. Energy-dispersive X-ray spectroscopy (EDX) confirmed reduced Ca/Si ratios, indicating enhanced calcium silicate hydrate (C-S-H) gel formation with superior binding characteristics. Results demonstrate that these previously unexploited rock powders effectively function as sustainable SCMs, reducing cement consumption by up to 12%, offering significant environmental benefits through reduced CO_2_ emissions and efficient utilization of natural geological resources in sustainable construction practices.

## 1. Introduction

Rocks have historically served fundamental roles in concrete technology, traditionally functioning as coarse and fine aggregates in structural applications [1,2,3]. Contemporary research has revealed that rocks can be processed into micro- and nano-sized powders, enabling their utilization as supplementary cementitious materials (SCMs) with significantly enhanced reactivity and performance characteristics [4,5]. Concrete, recognized as the world’s most widely consumed construction material after water, presents both significant opportunities and substantial challenges in addressing these sustainability concerns [6]. The production of ordinary Portland cement (OPC), concrete’s primary binding agent, contributes approximately 5% of global anthropogenic carbon dioxide emissions—a staggering environmental footprint that demands immediate mitigation strategies [7]. Researchers have explored various alternative materials and innovative approaches to reduce environmental impact, including geopolymer concrete systems [8,9], waste-derived aggregates [10], industrial by-products such as blast furnace slag [11], and bio-based enhancement strategies [12]. Beyond material substitution, studies have also demonstrated that the type and content of SCMs significantly influence early-age mechanical properties and deformation behaviors of cement-based systems, highlighting the importance of careful mix design optimization in sustainable concrete development [13].

The application of diverse rock powders as SCMs represents a critical advancement in sustainable construction technology. Silicate-rich rocks, encompassing compositions such as granite, rhyolite, and dacite, are characterized by elevated silica (SiO_2_) content. The critical component for pozzolanic activity is the presence of amorphous or cryptocrystalline silica phases, which exhibit exceptional reactivity within the alkaline environment of cement paste systems [14]. The amorphous silica component readily participates in pozzolanic reactions through consumption of calcium hydroxide [Ca(OH)_2_] generated during Portland cement hydration, forming additional secondary calcium silicate hydrate (C-S-H) gel—the primary binding phase responsible for concrete strength development [15]. The distinct reaction mechanisms suggest potential synergistic effects in binary or ternary SCM systems. Various naturally occurring rock powders, including granite, granodiorite, zeolite, rhyolite, pumice, and andesite, have been widely investigated as viable SCMs. Research demonstrates that zeolite exhibits improved performance when cement is partially replaced [16,17,18], pumice improves late-age strength at 25% replacement [19], and andesite maintains acceptable properties at 5–10% replacement [20,21,22].

The utilization of granodiorite in concrete has evolved from traditional aggregate applications to innovative cement replacement strategies, demonstrating its versatility as a construction material. Hussain et al. [23] evaluated granodiorite aggregates for frost durability, finding a durability factor of 98.4% after freeze–thaw testing, demonstrating excellent resistance to weathering and suitability for construction applications. Mahmoud et al. [24] investigated waste granodiorite powder (WGDP) as cement replacement, finding improved workability, accelerated hydration, and enhanced compressive strength at ambient and elevated temperatures, attributed to high fineness, void-filling capacity, and pozzolanic properties. Mahmoud et al. [25] reported that 7% WGDP replacement enhanced compressive strength by 24.7% at room temperature and 28% at 800 °C, while improving gamma-ray attenuation and fast neutron shielding. Fathy et al. [26] demonstrated optimal performance at 7% bulk granodiorite replacement with 19.1% compressive strength increase. Fathy et al. [27] compared micro- and nano-scale granodiorite, finding optimal ratios of 7% micro-GD and 4% nano-GD, with nano-scale particles exhibiting superior performance. Combined nano-GD with nano-PbO achieved synergistic effects, increasing compressive strength, tensile strength, and linear attenuation coefficient by 25.7%, 16.2%, and 44.7%, respectively. Fathy et al. [28] confirmed that 4% nano-granodiorite enhanced compressive strength by 17.3%. Mashaly et al. [29] showed that up to 20% granite sludge replacement exhibited minimal decline in physical and mechanical properties while enhancing resistance to abrasion, freeze–thaw cycles, and sulfate attack. Labbaci et al. [30] characterized six volcanic rocks (basalt, olivine andesite, amphibole-biotite andesite, amphibole andesite, rhyodacite, and scoria), demonstrating pozzolanic activity meeting ASTM C618 requirements with strength activity indices ranging from 82.11% to 91.91% at 28 days. Mortars containing 15% volcanic powder replacement demonstrated superior compressive strength. Abdelfattah et al. [31] showed meta basalt exhibited the highest compressive strength and bulk density with the lowest porosity due to compact microstructure. Lasheen et al. [32] confirmed Dokhan metavolcanics possess suitable pozzolanic properties for SCM applications with viable crushed aggregate potential.

### Research Significance

Despite growing interest in rock powder-based SCMs, the systematic evaluation of silicate- and aluminate-rich igneous and metavolcanic rock types sourced from Egypt’s largely unexploited Eastern Desert as individual cement replacement materials remains largely unexplored. GDP and AFGP represent felsic silicate-rich rock types with high combined SiO_2_ and Al_2_O_3_ contents, while MMVP represents a mafic aluminate- and iron-rich metavolcanic rock type—together spanning a broad compositional spectrum of Eastern Desert geological resources with significant yet untapped pozzolanic potential. Previous studies have predominantly focused on single rock types or mixed geological sources, leaving a critical knowledge gap regarding the comparative pozzolanic performance of these three compositionally distinct rock powders when used as individual SCMs. This study addresses this gap by processing Eastern Desert rock samples into ultrafine powders and systematically evaluating their performance as partial cement replacements. The experimental program encompasses pozzolanic activity quantification through the Strength Activity Index (SAI) in accordance with ASTM C311 [33], fresh concrete workability assessment, mechanical testing of compressive and splitting tensile strengths, and in-depth microstructural investigation using scanning electron microscopy (SEM) coupled with energy-dispersive X-ray spectroscopy (EDX) to examine hydration product formation, matrix densification, and interfacial characteristics at the microstructural level. This multi-faceted approach provides a scientifically rigorous framework for establishing these abundant yet underutilized Egyptian geological resources as viable, sustainable SCMs, contributing to reduced cement consumption and lower CO_2_ emissions in the construction industry.

## 2. Materials

### 2.1. Cement

The study employed ordinary Portland cement (OPC) Type I, Grade 42.5 N, complying with ASTM C 150 [34] specifications. Physical characterization included determination of specific gravity, surface area, standard consistency, and setting times (initial and final) to verify conformance with standard requirements. Mechanical performance was evaluated through compressive strength testing of mortar specimens at 3, 7, and 28 days of curing to establish strength development over time. Table 1 summarizes the physical properties and compressive strength progression of the cement. Chemical composition was determined using X-ray fluorescence (XRF) spectroscopy, which identified calcium and silica compounds as the primary constituents—essential components for cement hydration and strength development. The XRF results presented in Table 2 provide critical baseline information for understanding the cement’s chemical characteristics and expected performance behavior.

### 2.2. Aggregates

This study utilized natural siliceous sand as fine aggregate and crushed dolomite as coarse aggregate, with a maximum nominal size of 19 mm, conforming to ASTM C33/C33M [35]. The physical properties of the aggregates were characterized following established testing procedures, with findings presented in Table 3. Tests included specific gravity determined in accordance with ASTM C127 [36] for coarse aggregate and ASTM C128 [37] for fine aggregate, bulk density and void percentage assessed according to ASTM C29/C29M [38], absorption percentage following ASTM C127 and C128, and clay and fine material content evaluated using ASTM C117 [39] for both aggregate types. Type-specific tests were also conducted: the crushing value test was performed on the coarse aggregate in accordance with BS 812-110 [40] to evaluate its mechanical strength and resistance to crushing under compressive loads, while the fineness modulus was determined for the fine aggregate following ASTM C136/C136M [41] to characterize its particle size distribution and gradation.

### 2.3. Water and Superplasticizer

Mixing and curing of all concrete specimens were performed using potable water in accordance with ASTM C1602/C1602M [42] specifications. Sikament NN, a commercially available superplasticizer manufactured by Sika Company, Egypt, was utilized as a chemical admixture. The product meets the requirements of ASTM C494 [43].

### 2.4. Rock Powders

Rock samples were collected along the Marsa Alam–Idfu road in the southern part of the Eastern Desert of Egypt. The granodiorite was sampled at coordinates 25°04′20.52″ N and 34°17′57.07″ E, the alkali-feldspar granite at 25°02′42.97″ N and 34°33′12.57″ E, and the metavolcanic rocks at 25°03′39.95″ N and 34°48′40.84″ E. Petrographic investigations were carried out on thin sections of the rocks using a Leica DM750P polarized light microscope (Leica Microsystems, Wetzlar, Germany). The raw material underwent systematic mechanical processing: initial crushing via jaw crusher, followed by ball milling with steel balls for 4 h, and final sieving through ASTM No. 200 standard sieve (75 μm aperture) to ensure particle size uniformity suitable for SCM applications. The 75 μm sieve was used as a quality control step to ensure the complete removal of coarse particles and confirm that all powder particles passed below this threshold. The average particle size of 1.4–1.5 μm represents the measured particle size distribution of the processed powders, which is well within the sub-75 μm range confirmed by sieving.

#### 2.4.1. Granodiorite Powder (GDP)

**Figure 1:**
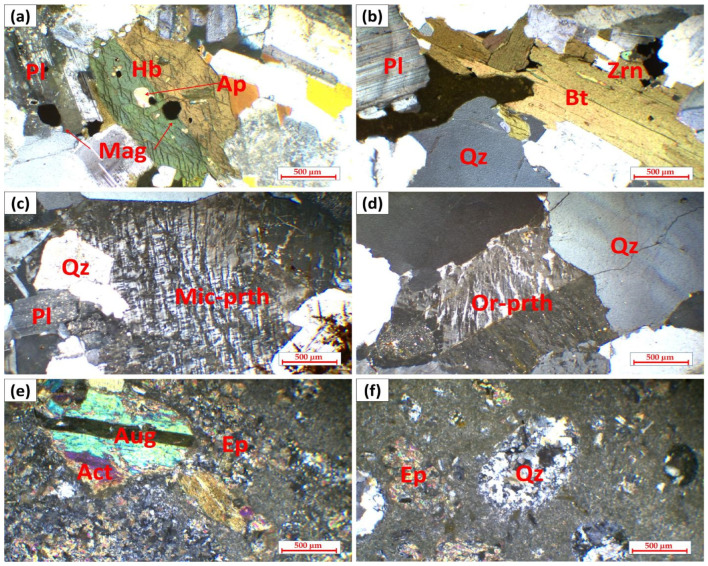
Microphotographs under crossed polarized light for the utilized rocks in concrete: (**a**) twinned hornblende (Hb) hosting apatite (Ap) and magnetite (Mag) in granodiorite, (**b**) quartz (Qz), plagioclase (Pl), and biotite (Bt) with accessory zircon (Zrn) components of granodiorite, (**c**) coarse perthitic microcline (Mic-prth) hosting smaller quartz and plagioclase in alkali-feldspar granite, (**d**) orthoclase perthite (Or-prth) and quartz components of alkali-feldspar granite, (**e**) twinned augite surrounded by epidote (ep) in fine groundmass in metavolcanics, (**f**) elliptical cavity filled with secondary quartz and irregular patches of epidote in metavolcanics.

**Figure 2:**
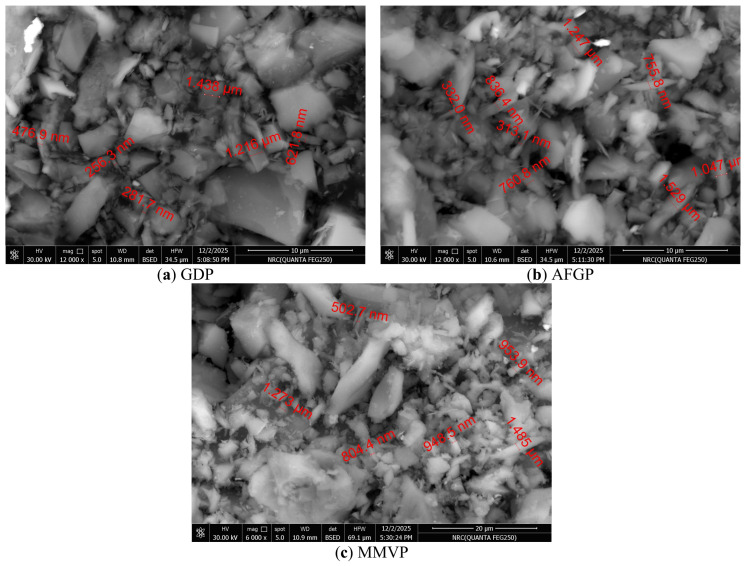
SEM images for: (**a**) GDP, (**b**) AFGP, and (**c**) MMVP.

Petrographic examination of the granodiorite reveals a coarse-grained, holocrystalline texture dominated by sodic plagioclase feldspar, principally of andesine–oligoclase composition, which constitutes more than 50% of the total feldspar content (Figure 1a,b). Plagioclase crystals commonly exhibit well-developed compositional zoning and polysynthetic (albite) twinning (Figure 1b), and are locally affected by sericitization and epidote alteration along cleavage planes. Potassium feldspar occurs in minor amounts as small orthoclase grains displaying simple twinning. Quartz is abundant and present as subhedral to anhedral grains, typically occupying interstitial spaces between feldspar crystals. Under crossed polars, quartz displays undulose extinction, indicative of mild deformation during emplacement or subsequent tectonothermal events. Hornblende is a prominent ferromagnesian phase, commonly forming well-developed, euhedral to subhedral prismatic grains with characteristic six-sided, diamond-shaped basal sections and two sets of cleavage intersecting at approximately 120°. Simple twinning is locally observed in some hornblende grains (Figure 1a). Hornblende crystals frequently host accessory inclusions, including magnetite and apatite. Biotite occurs as coarse, pleochroic greenish-brown flakes with pronounced basal cleavage. Partial chloritization along cleavage planes is observed in some grains. Biotite locally contains inclusions of zircon and magnetite (Figure 1b). XRF analysis (Table 2) revealed chemical composition dominated by SiO_2_ (68.98%) and Al_2_O_3_ (15.71%), with notable Fe_2_O_3_ (3.52%), Na_2_O (3.14%), CaO (3.18%), and K_2_O (2.08%). The sum of principal oxides (SiO_2_ + Al_2_O_3_ + Fe_2_O_3_ = 88.21%) significantly exceeds the 70% minimum specified by ASTM C618 for natural pozzolans [44]. SEM analysis (Figure 2a) revealed irregularly shaped, angular particles with average size of 1.4 μm, typical of mechanically fractured crystalline materials. Physical properties including specific gravity (2.70), loss on ignition (0.91%), and SO_3_ content (0.08%) are summarized in Table 4, all meeting ASTM C618 requirements for natural pozzolans.

#### 2.4.2. Alkali Feldspar Granite Powder (AFGP)

The alkali-feldspar granite is markedly enriched in potassium feldspar and quartz, which together constitute more than 90 vol.% of the rock (Figure 1c,d). Potassium feldspar is the dominant phase, occurring primarily as orthoclase (Figure 1c) and microcline perthite (Figure 1d), and typically forms large, blocky crystals with pink to flesh-colored hues. Perthitic textures, produced by the exsolution of albite lamellae within K-feldspar during slow subsolidus cooling, are well developed and range from coarse to finely intergrown varieties. Quartz is abundant and occurs mainly as anhedral to subhedral grains with interlocking boundaries. Locally, graphic intergrowths between quartz and alkali feldspar are observed, indicating crystallization from a residual, silica-rich melt during the late stages of magmatic differentiation. Plagioclase is subordinate in abundance (Figure 1c) and is predominantly sodic in composition (albite to oligoclase). These grains commonly exhibit extensive sericitization and may display simple or polysynthetic twinning, although compositional zoning is generally absent. Muscovite occurs in minor amounts as small flakes occupying interstitial spaces between the blocky feldspar crystals. Mafic minerals are scarce and are entirely replaced by secondary chlorite and hematite, imparting a characteristic reddish discoloration to the rock. Accessory phases are relatively abundant and include zircon, fluorite, allanite, monazite, titanite, and apatite, collectively reflecting the highly evolved, incompatible-element-enriched character of the alkali-feldspar granite. XRF analysis (Table 2) revealed a chemical composition with the highest SiO_2_ content (73.23%) among the tested materials, accompanied by Al_2_O_3_ (13.91%), K_2_O (5.86%), and Na_2_O (2.78%), with minimal Fe_2_O_3_ (1.47%) and CaO (0.91%). Notably, the K_2_O content (5.86%) exceeds twice the Na_2_O content, confirming the alkali feldspar granite classification according to petrographic nomenclature. The sum of principal oxides (SiO_2_ + Al_2_O_3_ + Fe_2_O_3_ = 88.61%) significantly exceeds the 70% minimum specified by ASTM C618 for natural pozzolans. SEM analysis (Figure 2b) revealed irregularly shaped, angular particles with an average size of 1.5 μm, morphology characteristic of mechanically fractured crystalline materials. Physical properties including specific gravity (2.62), loss on ignition (0.88%), and SO_3_ content (0.05%) are summarized in Table 4, all meeting ASTM C618 requirements for natural pozzolans.

#### 2.4.3. Mafic Metavolcanic Powder (MMVP)

The mafic metavolcanic rocks, compositionally equivalent to basaltic andesite, recorded low- to medium-grade metamorphism reaching greenschist to lower amphibolite facies, while preserving diagnostic primary igneous features of the volcanic protolith overprinted by metamorphic recrystallization. Mineralogically, these rocks are characterized by relict plagioclase feldspar phenocrysts that are variably preserved and partially replaced by fine-grained assemblages of albite, epidote, and sericite. Relict augite crystals are locally retained but are commonly rimmed or pseudomorphed by actinolite along their margins (Figure 1e). Actinolite is the dominant metamorphic amphibole, occurring as prismatic to fibrous crystals that are commonly aligned parallel to the rock fabric and display pale to dark green pleochroism. Chlorite is a ubiquitous secondary phase, forming after amphibole and biotite, and imparts a characteristic green coloration to the rock. Epidote is a prominent accessory mineral, present as granular to prismatic crystals disseminated throughout the matrix or concentrated along fractures and cleavage planes within relict augite grains (Figure 1f). Quartz occurs in both primary and secondary forms. Primary quartz is present in minor amounts as anhedral interstitial grains within the groundmass, whereas secondary quartz commonly fills elliptical vesicles and amygdales inherited from the volcanic protolith (Figure 1f). Opaque minerals, chiefly magnetite and ilmenite, are abundant and locally exhibit partial oxidation to hematite. Additional accessory phases include apatite and titanite, which likely formed during metamorphic reactions involving Ca-bearing minerals. Texturally, the rocks commonly preserve a relict porphyritic fabric, with original plagioclase phenocrysts set within a fine- to medium-grained metamorphic groundmass. The groundmass exhibits granoblastic to nematoblastic textures, reflecting recrystallization under greenschist to lower amphibolite facies conditions. The preferred orientation of actinolite and chlorite defines a weak to moderate schistosity, particularly in more intensely deformed samples. Vesicular to amygdaloidal textures are locally preserved, with vesicles infilled by secondary minerals such as quartz, calcite, chlorite, and epidote. XRF analysis (Table 2) revealed a distinctly different chemical composition compared to the felsic powders, characterized by moderate SiO_2_ (52.82%), elevated Al_2_O_3_ (15.25%), and notably high Fe_2_O_3_ (10.37%) and MgO (7.76%), reflecting the mafic nature of the parent rock. Additional components include CaO (5.99%), Na_2_O (3.82%), and minimal K_2_O (0.76%). The sum of principal oxides (SiO_2_ + Al_2_O_3_ + Fe_2_O_3_ = 78.44%) exceeds the 70% minimum specified by ASTM C618 for natural pozzolans. SEM analysis (Figure 2c) revealed irregularly shaped, angular particles with an average size of 1.5 μm, morphology characteristic of mechanically fractured crystalline materials. The metamorphic mineral assemblage (actinolite-chlorite-plagioclase) indicates greenschist facies metamorphism of basaltic protolith. Physical properties including specific gravity (2.79—the highest among tested materials), loss on ignition (1.93%), and absence of detectable SO_3_ are summarized in Table 4, all meeting ASTM C618 requirements for natural pozzolans.

## 3. Experiments

### 3.1. Mix Design and Mixing Procedures

The experimental program comprised 13 concrete mixtures, consisting of one control mixture and 12 modified compositions incorporating varying percentages of three distinct types of rock powders (GDP, AFGP, and MMVP) as partial cement replacement. The detailed mix proportions of concrete are presented in Table 5. The replacement range of 3–12% was selected based on levels commonly reported in studies investigating natural rock powders as SCMs in concrete [25,26]. The upper bound of 12% was intentionally chosen to encompass the expected zone of performance decline beyond the optimal replacement level, enabling systematic identification of the optimal threshold for each rock powder rather than simply confirming feasibility at a fixed replacement level. The incremental step of 3% between consecutive replacement levels was adopted to provide adequate resolution for capturing performance trends and precisely locating the optimal replacement threshold for GDP, AFGP, and MMVP.

### 3.2. Casting and Curing

Following the completion of the mixing procedure, the fresh concrete was systematically cast into standardized steel molds of two geometric configurations: cubic specimens measuring 100 × 100 × 100 mm for the determination of compressive strength, and cylindrical specimens with a diameter of 100 mm and height of 200 mm for the assessment of splitting tensile strength, in accordance with ASTM C192/C192M [45]. Following the initial mold retention period, the specimens were carefully demolded and immediately transferred to a controlled aqueous curing environment in accordance with ASTM C31/C31M [46]. The curing regime was conducted in potable water maintained at a constant temperature of 23 ± 2 °C.

### 3.3. Test Methods

#### 3.3.1. Strength Activity Index (SAI)

GDP, AFGP, and MMVP were confirmed as suitable pozzolans per ASTM C618, with combined oxide contents (SiO_2_ + Al_2_O_3_ + Fe_2_O_3_) of 88.21%, 88.61%, and 78.44%, respectively, all exceeding the required 70% minimum. To quantify their pozzolanic performance, the Strength Activity Index (SAI) was evaluated at 28 days following ASTM C311. Four mortar mixtures were prepared: one control mix (MO) with 100% OPC, and three test mixes with 20% cement replaced by GDP, AFGP, and MMVP, respectively. Specimens were cured in a moist room at 23 ± 2 °C for 24 h, then demolded and immersed in saturated water until testing. The SAI was calculated as (A/B) × 100, where A and B are the 28-day compressive strengths of the test mix and the control mix (MO), respectively. A minimum SAI of 75% is required by ASTM C618 for pozzolanic classification.

#### 3.3.2. Workability

To evaluate the influence of different rock powders (GDP, AFGP, and MMVP) on fresh concrete properties, workability was quantified through the slump cone test following ASTM C143/C143M [47], a standardized and widely recognized measure of flowability, cohesion, and ease of placement in field applications. Tests were conducted immediately following the completion of mixing using a standardized slump cone (top diameter 100 mm, base diameter 200 mm, height 300 mm), thereby providing quantitative evidence of the differential effects of GDP, AFGP, and MMVP on concrete workability and consistency.

#### 3.3.3. Mechanical Properties

To study the effect of GDP, AFGP, and MMVP as cement replacement materials in single mixtures, a comprehensive experimental investigation was undertaken to evaluate the mechanical performance of all concrete mixtures through standardized testing methodologies. All mechanical property evaluations were conducted utilizing a calibrated compression testing machine with a nominal capacity of 3000 KN, ensuring consistent load application, precise measurement accuracy, and reproducible results. Compressive strength was evaluated on cubic specimens measuring 100 × 100 × 100 mm in accordance with ASTM C39/C39M [48] at curing ages of 7, 28, and 90 days to characterize both early-age and long-term strength performance. Uniaxial compressive loading was applied at a controlled rate of strain until specimen failure. For each mixture composition and curing age, a minimum of three replicate specimens were tested, and reported values represent the arithmetic mean, thereby providing statistically meaningful data and minimizing specimen variability. Splitting tensile strength was evaluated in accordance with ASTM C496/C496M [49] using cylindrical specimens (100 mm diameter × 200 mm height) at 28 days of curing.

#### 3.3.4. Microstructural Analysis

A comprehensive microstructural investigation was systematically conducted utilizing field emission scanning electron microscopy (FE-SEM) coupled with energy dispersive X-ray spectroscopy (EDX) in accordance with ASTM C1723 [50] to evaluate the influence of GDP, AFGP, and MMVP incorporation on concrete matrix morphology and phase composition. Representative specimens (10 × 10 mm) were extracted from concrete cube cores after compressive testing and dried at 60 °C for 24 h to arrest hydration.

## 4. Experimental Results

### 4.1. SAI (%)

The 28-day compressive strengths of the control mortar (MO) and the three test mixes were used to calculate the SAI for each rock powder in accordance with ASTM C311. All three rock powders exceeded the minimum SAI threshold of 75% required by ASTM C618, confirming their classification as active pozzolanic materials. The SAI values were 82%, 79%, and 76% for AFGP, GDP, and MMVP, respectively. The highest SAI was recorded for AFGP, consistent with its highest combined oxide content (88.61%), followed by GDP (88.21%) and MMVP (78.44%), reflecting a direct correlation between chemical composition and pozzolanic reactivity. Although MMVP recorded the lowest SAI among the three materials, it still surpassed the 75% minimum threshold, confirming its validity as a pozzolanic SCM. These results validate that the ultrafine rock powders contribute not only through physical filler effects but also through pozzolanic reactivity, forming additional C-S-H gel that densifies the mortar matrix and enhances mechanical performance.

### 4.2. Slump Test Result

**Figure 3:**
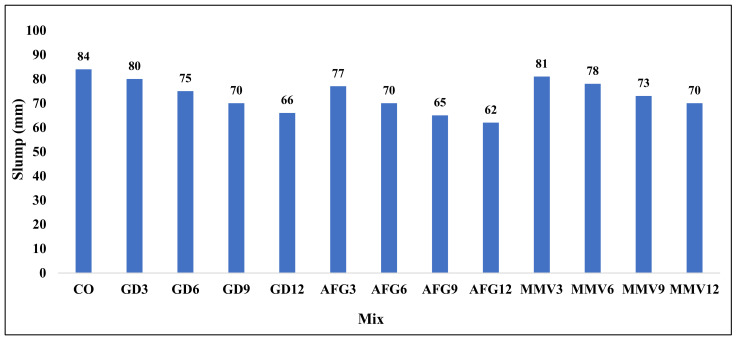
Slump test results for all concrete mixes.

As illustrated in Figure 3, the slump cone test results for all concrete mixes were obtained immediately after mixing and before casting to assess the fresh concrete workability. The control mix (CO) achieved a slump value of 84 mm, establishing the baseline workability for comparison with modified mixtures. The incorporation of all three rock powders (GDP, AFGP, and MMVP) resulted in progressive slump reduction as the cement replacement percentage increased, indicating decreased workability with higher content. The GDP replacement results demonstrated a clear trend of workability reduction proportional to the replacement level. The GD3, GD6, GD9, and GD12 mixes recorded slump values of 80, 75, 70, and 66 mm, respectively, corresponding to decreases of 4.8%, 10.7%, 16.7%, and 21.4% compared to the control. This systematic reduction in slump is primarily attributed to the significant differences in particle size and surface area between OPC and GDP. The observed slump reduction is in agreement with previous investigations, which reported similar workability decreases when GDP was used as a partial cement replacement [27]. Similarly, AFGP powder exhibited comparable physical characteristics with an average particle size of 1.5 µm and a surface area of 11,800 cm^2^/g. The AFGP series showed slump values of 77, 70, 65, and 62 mm for AFG3, AFG6, AFG9, and AFG12, respectively, representing reductions of 8.3%, 16.7%, 22.6%, and 26.2% compared to the control. The slightly less severe workability reduction compared to GDP, despite similar particle size, can be attributed to the marginally lower surface area of AFGP. The MMVP, with an average particle size of 1.5 µm and surface area of 12,100 cm^2^/g, demonstrated the most pronounced effect on workability. The MMV series recorded slump values of 81, 78, 73, and 70 mm for MMV3, MMV6, MMV9, and MMV12, respectively, corresponding to reductions of 3.6%, 7.1%, 13.1%, and 16.7%. The observed workability reductions, while notable, remained within acceptable ranges for conventional concrete placement methods. All mixes maintained slump values above 60 mm, which is generally considered adequate for standard construction applications.

### 4.3. Mechanical Properties Results

#### 4.3.1. Compressive Strength

**Figure 4:**
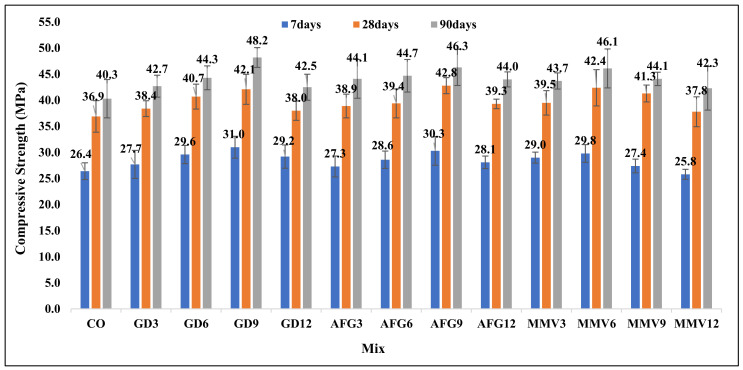
Compressive strength for all concrete mixes.

Figure 4 and Table 6 show the compressive strength results at 7, 28, and 90 days. The control mix (CO) achieved baseline strengths of 26.4 MPa, 36.9 MPa, and 40.3 MPa at 7, 28, and 90 days, respectively. At 7 days, the GDP replacement series exhibited progressive strength enhancement with increasing replacement levels up to 9%. The GD3, GD6, GD9, and GD12 mixes achieved compressive strengths of 27.7, 29.6, 31.0, and 29.2 MPa, corresponding to increases of 4.9%, 12.1%, 17.4%, and 10.6%, respectively. At 28 days, GDP performance improved further, with GD3, GD6, GD9, and GD12 achieving 38.4, 40.7, 42.1, and 38.0 MPa, representing enhancements of 4.1%, 10.3%, 14.1%, and 3.0%. At 90 days, the GDP continued to gain strength, with GD3, GD6, GD9, and GD12 reaching 42.7, 44.3, 48.2, and 42.5 MPa, corresponding to increases of 6.0%, 10.2%, 19.6%, and 5.5% over the control, with GD9 achieving the highest 90-day strength among all GDP mixes. The optimal GDP replacement was 9% at both ages. These results align with previous investigations. The AFGP results demonstrated similar trends, with AFG3, AFG6, AFG9, and AFG12 achieving 27.3, 28.6, 30.3, and 28.1 MPa at 7 days (increases of 3.4%, 8.3%, 14.8%, and 6.4%), and 38.9, 39.4, 42.8, and 39.3 MPa at 28 days (improvements of 5.4%, 6.8%, 16.0%, and 6.5%). At 90 days, AFG3, AFG6, AFG9, and AFG12 reached 44.1, 44.7, 46.3, and 44.0 MPa, representing gains of 9.4%, 10.9%, 14.9%, and 9.2% over the control, confirming that pozzolanic reactions continued to strengthen these mixes at extended curing ages. The optimal AFGP replacement was 9%, achieving the highest absolute strength among all mixtures at both 28 days (42.8 MPa) and 90 days (46.3 MPa). The MMVP results exhibited more variable performance. At 7 days, MMV3, MMV6, MMV9, and MMV12 achieved 29.0, 29.8, 27.4, and 25.8 MPa (changes of +9.8%, +12.9%, +3.8%, and −2.3%), with optimal performance at 6% replacement. At 28 days, these mixes achieved 39.5, 42.4, 41.3, and 37.8 MPa (improvements of 7.0%, 14.9%, 11.9%, and 2.4%), maintaining optimal performance at 6%. At 90 days, MMV3, MMV6, MMV9, and MMV12 reached 43.7, 46.1, 44.1, and 42.3 MPa, corresponding to increases of 8.4%, 14.4%, 9.4%, and 5.0% over the control, with MMV6 (46.1 MPa) retaining its position as the optimal MMVP replacement level across all curing ages. The compressive strength improvements achieved at the optimal replacement levels of 9% for GDP and AFGP, and 6% for MMVP (14.1%, 16.0%, and 14.9% at 28 days, respectively) are consistent with and comparable to strength improvements reported for well-established SCMs such as silica fume at its optimal 10% replacement level, where superior strength was attributed to particle fineness and improved particle packing effects [51]. This confirms that the ultrafine particle size and pozzolanic reactivity of GDP, AFGP, and MMVP produce mechanical performance enhancements comparable to those of conventional SCMs, while offering the additional advantage of utilizing locally abundant Eastern Desert geological resources.

#### 4.3.2. Splitting Tensile Strength

**Figure 5:**
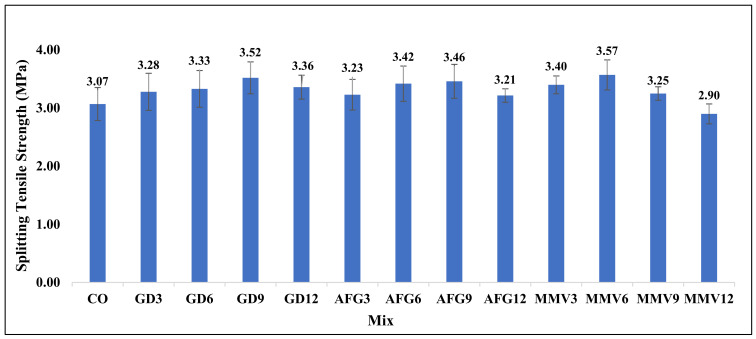
Splitting tensile strength at 28 days: results for all concrete mixes.

As illustrated in Figure 5 and Table 6, the splitting tensile strength results at 28 days demonstrate the ability of pozzolanic rock powders to enhance the tensile capacity of concrete when used as partial cement replacements. The control concrete sample (CO) achieved a baseline splitting tensile strength of 3.07 MPa. The incorporation of all three rock powders generally resulted in improved tensile strength across most replacement dosages, confirming the beneficial effects of these SCMs on both compressive and tensile properties. The GDP mixes exhibited progressive enhancement in splitting tensile strength with increasing replacement levels. The GD3, GD6, GD9, and GD12 mixes achieved tensile strengths of 3.28, 3.33, 3.52, and 3.36 MPa, respectively, corresponding to improvements of 6.8%, 8.5%, 14.7%, and 9.4% compared to the control. The optimal performance was clearly observed at 9% replacement (GD9), which demonstrated the highest tensile strength among the GDP series. Although the strength slightly decreased at 12% replacement (GD12), it remained significantly higher than the control, indicating that GDP maintains beneficial effects on tensile properties even at elevated replacement levels. These results correlate well with both the 7-day and 28-day compressive strength trends, where GD9 also exhibited optimal performance. The splitting tensile strength improvements align with previous investigations [24,27] that documented enhanced tensile capacity in GDP-modified concrete. As mentioned before, the enhancement can be attributed to the refined microstructure resulting from C-S-H and C-A-H gel formation through pozzolanic reactions, combined with the densification of the interfacial transition zone due to GDP’s high specific surface area and ultrafine particle size. Similarly, the AFGP mixes demonstrated good tensile strength performance. The AFG3, AFG6, AFG9, and AFG12 mixes achieved splitting tensile strengths of 3.23, 3.42, 3.46, and 3.21 MPa, representing improvements of 5.2%, 11.4%, 12.7%, and 4.8% over the control, respectively. The optimal replacement level for AFGP was 9%, where AFG9 achieved 3.46 MPa, though the improvement was slightly less pronounced than that observed for GDP at the same replacement level. Nevertheless, AFGP demonstrated consistent enhancement across all replacement dosages, with even the 12% replacement maintaining meaningful improvement, suggesting good compatibility between AFGP and Portland cement in terms of tensile load transfer mechanisms. The MMVP mixes exhibited the most variable tensile strength performance. The MMV3, MMV6, MMV9, and MMV12 mixes achieved splitting tensile strengths of 3.40, 3.57, 3.25, and 2.90 MPa, corresponding to changes of +10.7%, +16.3%, +5.9%, and −5.5%, respectively. Remarkably, MMV6 demonstrated the highest splitting tensile strength among all tested mixtures (3.57 MPa), representing a 16.3% improvement over the control. However, the performance declined sharply at higher replacement levels, with MMV12 falling below the control strength. This pattern mirrors the compressive strength results and confirms that MMVP’s optimal replacement level is restricted to 6% for maintaining both compressive and tensile properties. The correlation between splitting tensile strength and compressive strength followed the expected relationship for all mixes. The proportional improvements in both compressive and tensile properties suggest that the pozzolanic reactions not only increase the overall strength but also enhance the bond quality at the aggregate–paste interface, which is critical for tensile load resistance.

### 4.4. Microstructural Analysis Results

#### 4.4.1. SEM Analysis

**Figure 6:**
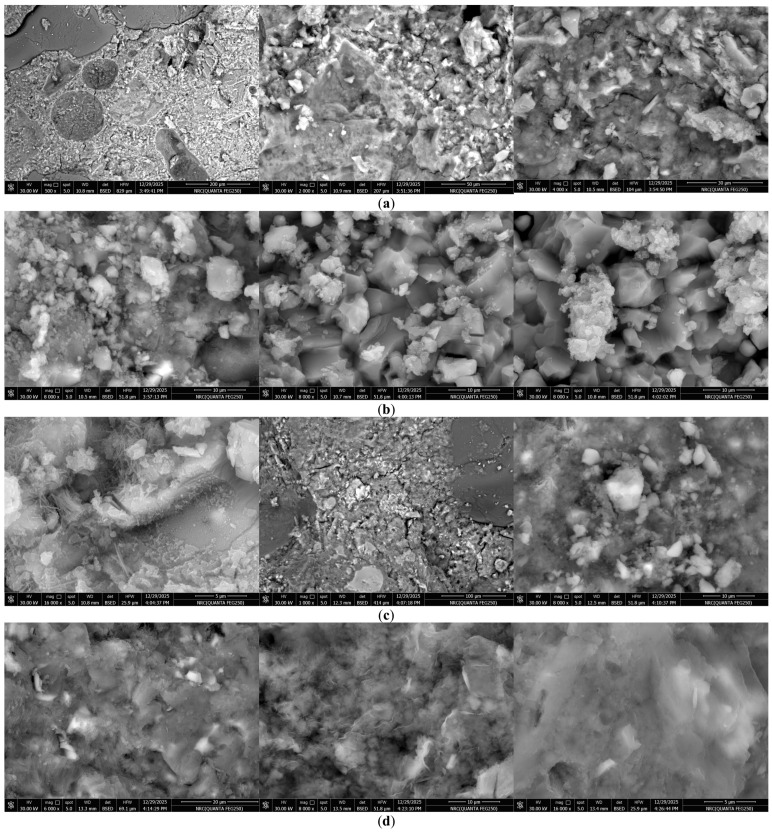
SEM images for concrete samples: (**a**) CO, (**b**) GD9, (**c**) AFG9, and (**d**) MMV6.

SEM analysis was conducted on the control concrete sample (CO) and three optimum samples (GD9, AFG9, and MMV6) selected based on superior mechanical performance. Figure 6 presents the SEM images of the control and optimum samples at various magnifications. Figure 6a displays the SEM images of the control sample CO at magnifications of 500×, 2000×, and 4000×, which exhibited a relatively poor microstructure characterized by numerous pores and cracks of varying sizes, ranging from small microcracks to wider fissures. The matrix appeared less homogeneous and poorly compacted, with a loosely packed structure and high porosity, indicating suboptimal particle packing and limited hydration product formation. In contrast, concrete samples containing GD9, AFG9, and MMV6 demonstrated significant microstructural improvements, as shown in Figure 6b–d. Figure 6b presents SEM images of GD9 at 8000× magnification, revealing a noticeably denser matrix with improved particle packing and reduced visible porosity compared to the control. Figure 6c presents SEM images of AFG9 at magnifications of 16,000×, 1000×, and 8000×, showing a compact microstructure with well-distributed hydration products and a denser interfacial transition zone (ITZ). Figure 6d presents SEM images of MMV6 at 6000×, 8000×, and 16,000× magnifications, demonstrating the densest matrix among the optimum samples, consistent with the superior strength improvement recorded for MMV6. The SEM observations across all optimum samples revealed abundant hydration products, particularly C-S-H gel, which is the primary binding phase in concrete [52]. The observed microstructural improvements are consistent with the mechanical performance enhancements recorded at 28 days, where compressive strength improvements of 14.1%, 16.0%, and 14.9% were achieved for GD9, AFG9, and MMV6, respectively, suggesting that the denser matrix and reduced porosity observed in SEM images are associated with improved mechanical performance. However, it is acknowledged that a direct causal link between the observed microstructural features and mechanical performance cannot be established from SEM observations alone, as the relative contributions of physical filler effects and pozzolanic reactivity cannot be quantitatively isolated from qualitative SEM analysis. These observations are therefore presented as correlative evidence, consistent with the SAI results, which collectively support the role of pozzolanic reactivity and physical filler effects in enhancing matrix densification and binding capacity [53,54]. This densification and increased hydration product formation directly correlate with the improved mechanical properties observed in these optimum samples, validating the effectiveness of these rock powders as sustainable cement replacement materials [55]. The formation of additional C-S-H gel through pozzolanic reactions effectively fills the interfacial voids, improving matrix continuity and reducing the weakening effect typically associated with the ITZ, a mechanism similarly reported in repair material studies where enhanced chemical bonding within the ITZ compensated for microstructural weaknesses [56].

#### 4.4.2. EDX Analysis

**Figure 7:**
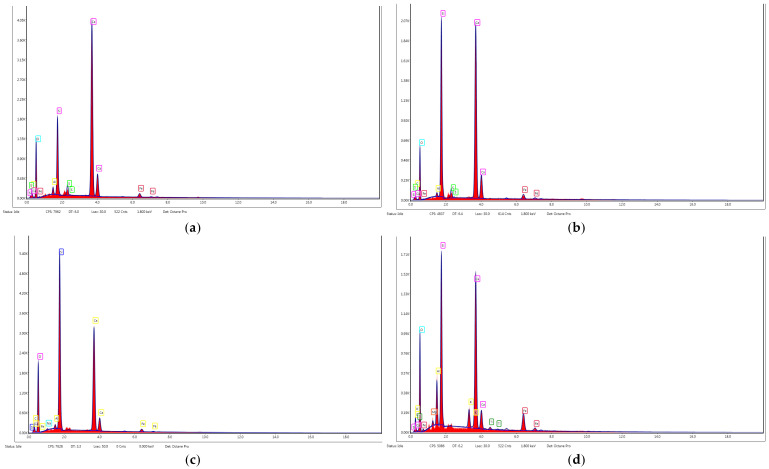
EDX patterns for concrete samples: (**a**) CO, (**b**) GD9, (**c**) AFG9, and (**d**) MMV6.

EDX analysis was performed on the control sample and three optimum concrete samples to investigate their elemental compositions and correlate them with mechanical performance. Figure 7 and Table 7 present the elemental composition by weight percentage for all tested samples. The Ca/Si ratios calculated from EDX data were 2.89, 1.37, 1.23, and 1.13 for CO, GD9, AFG9, and MMV6, respectively. The corresponding 28-day compressive strengths were 42.1, 42.8, and 42.4 MPa for GD9, AFG9, and MMV6, respectively, compared to the control sample CO. This progressive reduction in Ca/Si ratio is consistent with the trend of increasing compressive strength across the optimum samples, supporting the inverse relationship between Ca/Si ratio and mechanical performance widely reported in the literature [24,28,57,58,59,60]. Specifically, as the Ca/Si ratio decreased from 2.89 in CO to 1.37, 1.23, and 1.13 in GD9, AFG9, and MMV6, respectively, a corresponding improvement in compressive strength was observed, suggesting that the incorporation of rock powders promoted the formation of C-S-H gel with lower Ca/Si ratios and denser structure with improved binding capacity. These findings are further corroborated by the SAI results, which confirmed pozzolanic reactivity for all three rock powders with values of 79%, 82%, and 76% for GDP, AFGP, and MMVP, respectively, all exceeding the 75% minimum threshold required by ASTM C618. The consistent ranking observed across SAI values, Ca/Si ratios, and compressive strength results collectively reinforces the role of pozzolanic reactivity in driving the microstructural and mechanical improvements recorded for the optimum samples. Additionally, Table 7 reveals distinctive elemental signatures in the MMV6 sample, particularly elevated iron (Fe) content (3.42 wt%) compared to other samples, directly attributed to the high iron oxide content (Fe_2_O_3_ = 10.37%) in MMV rock powder. Similarly, the presence of magnesium (Mg K = 0.64 wt%) in MMV6 corresponds to the significant magnesium oxide content (MgO = 7.76%) in MMV.

## 5. Conclusions and Future Studies

This investigation systematically evaluated three distinct rock powders—granodiorite (GDP), alkali-feldspar granite (AFGP), and mafic metavolcanic (MMVP)—as sustainable supplementary cementitious materials in concrete. Based on the comprehensive experimental program encompassing workability, mechanical properties, and microstructural analyses, the following conclusions are drawn:

1. All three rock powders demonstrated favorable pozzolanic characteristics, with combined oxide contents (SiO_2_ + Al_2_O_3_ + Fe_2_O_3_) of 88.21%, 88.61%, and 78.44% for GDP, AFGP, and MMVP, respectively, significantly exceeding ASTM C618 requirements for natural pozzolans. These values, coupled with SAI values of 79%, 82%, and 76% for GDP, AFGP, and MMVP, respectively—all surpassing the 75% minimum threshold—confirm that chemical composition alone is insufficient for pozzolanic classification, and that mechanical activity indices provide essential complementary validation.
2. Workability progressively decreased with increasing rock powder content due to their ultrafine particle sizes (1.4–1.5 μm) and elevated specific surface areas (11,800–13,050 cm^2^/g), though all mixtures maintained acceptable slump values above 60 mm for practical construction applications. This trade-off between workability and reactivity is an inherent characteristic of ultrafine SCMs and should be considered in mix design optimization, particularly for applications requiring high flowability.
3. The optimal replacement levels varied by powder type: 9% for GDP and AFGP, and 6% for MMVP—a difference attributed to the lower combined oxide content and higher MgO and Fe_2_O_3_ content of MMVP, which limits its optimal dosage compared to the more felsic GDP and AFGP. At these dosages, compressive strength improvements of 14.1%, 16.0%, and 14.9% were achieved at 28 days, while splitting tensile strength increased by 14.7%, 12.7%, and 16.3%, respectively, compared to the control mixture. These improvements are comparable to those reported for well-established SCMs such as Class F fly ash and natural pozzolans, confirming the competitive performance potential of Eastern Desert rock powders.
4. SEM analysis revealed that rock powder incorporation substantially improved microstructural characteristics through dual mechanisms: physical filler effects reducing porosity and void content, and chemical pozzolanic reactions generating additional C-S-H gel formation, resulting in denser, more homogeneous matrices.
5. EDX analysis confirmed reduced Ca/Si ratios (1.37, 1.23, and 1.13 for GD9, AFG9, and MMV6 versus 2.89 for control), consistent with enhanced C-S-H gel formation in pozzolanic systems. The inverse correlation between Ca/Si ratio and compressive strength is corroborated by the SAI results, collectively providing multi-scale evidence of pozzolanic reactivity.

## 6. Limitations and Future Studies

While the present study establishes the fundamental physical, mechanical, and microstructural performance of concrete incorporating GDP, AFGP, and MMVP as SCMs, several limitations are acknowledged. The sustainability claims are primarily limited to cement consumption reduction and associated CO_2_ emissions mitigation. Durability metrics, long-term performance beyond 28 days, life-cycle assessment, grinding energy analysis, and raw material variability across multiple geological sources were beyond the scope of the present investigation. Additionally, only single-source materials were utilized, and the natural compositional variability of these rock types across different quarry locations within Egypt’s Eastern Desert was not addressed, which may influence pozzolanic performance under field conditions.

Future studies should address the long-term durability of GDP-, AFGP-, and MMVP-blended concrete, including chloride ion penetration resistance, carbonation, sulfate attack, alkali-silica reaction, and freeze–thaw performance. Water absorption, sorptivity, permeability, shrinkage, and creep behavior should also be evaluated to provide a comprehensive durability profile. The modulus of elasticity and Poisson’s ratio should be determined for complete mechanical characterization. Advanced analytical techniques including thermogravimetric analysis (TGA), isothermal calorimetry, and mercury intrusion porosimetry (MIP) are recommended to provide deeper insights into hydration kinetics and pore structure evolution of these rock powder-blended systems. Furthermore, multi-source variability studies examining GDP, AFGP, and MMVP from different Eastern Desert locations are encouraged to assess compositional consistency and its effect on concrete performance. Finally, field-scale validation studies combined with a comprehensive life-cycle assessment (LCA), grinding energy analysis, and economic feasibility evaluation are necessary to fully establish these Eastern Desert rock powders as viable SCMs for large-scale sustainable construction applications.

## Figures and Tables

**Table 1:** Properties of OPC.

Physical Properties							Mortar Compressive Strength (MPa)		
Color	Specific gravity	Initial setting time (min)	Final setting time (min)	Standard consistency (w/c%)	Average particle size (μm)	Surface area (cm^2^/g)	3 days	7 days	28 days
Grey	3.15	110	190	28%	18	3050	26	34	44

**Table 2:** Chemical composition of cement and rock powders (% mass).

	SiO_2_	CaO	Al_2_O_3_	Fe_2_O_3_	MgO	SO_3_	Na_2_O	K_2_O	P_2_O_5_	Mn_2_O_3_	TiO_2_	LOI *
**OPC**	21.72	60.59	4.85	1.91	2.27	2.8	0.56	0.42	0.22	0.17	0.55	3.15
**GDP**	68.98	3.18	15.71	3.52	1.58	0.08	3.14	2.08	0.08	0.09	0.51	0.91
**AFGP**	73.23	0.91	13.91	1.47	0.55	0.05	2.78	5.86	0.11	0.03	0.18	0.88
**MMVP**	52.82	5.99	15.25	10.37	7.76	-----	3.82	0.76	0.30	0.22	0.78	1.93

* LOI: Loss on ignition at 1000 °C.

**Table 3:** Physical properties of the used aggregates.

Property	Specific Gravity	Bulk Density (kg/m^3^)	Voids (%)	Absorption (%)	Clay and Fine Materials (%)	Crushing Value (%)	Fineness Modulus
Fine Aggregate	2.5	1730	30.8	1.5	1	--	2.27
Coarse Aggregate	2.6	1430	45	1.5	0.5	24	--

**Table 4:** Properties of powders and ASTM C618 requirement for natural pozzolan.

Property	GDP	AFGP	MMVP	ASTM C618 Requirement for Natural Pozzolan
(SiO_2_ + Al_2_O_3_ + Fe_2_O_3_) %	88.21	88.61	78.44	minimum = 70
SO_3_%	0.08	0.05	----	maximum = 4
Loss on ignition%	0.91	0.88	1.93	maximum = 10
Color	Light gray	Light pink	Gray	----
Specific gravity	2.70	2.62	2.79	----
Average particle size (µm)	1.4	1.5	1.5	----
Surface area (cm^2^/g)	13,050	11,800	12,100	----

**Table 5:** Concrete mix design (kg/m^3^).

Mix	Symbol	Cement	GDP	AFGP	MMVP	Aggregate		Water	SP
						Coarse	Fine		
Control	CO	400	----	----	----	1200	620	160	5
3% GDP	GD3	388	12	----	----	1200	620	160	5
6% GDP	GD6	376	24	----	----	1200	620	160	5
9% GDP	GD9	364	36	----	----	1200	620	160	5
12% GDP	GD12	352	48	----	----	1200	620	160	5
3% AFGP	AFG3	388	----	12	----	1200	620	160	5
6% AFGP	AFG6	376	----	24	----	1200	620	160	5
9% AFGP	AFG9	364	----	36	----	1200	620	160	5
12% AFGP	AFG12	352	----	48	----	1200	620	160	5
3% MMVP	MV3	388	----	----	12	1200	620	160	5
6% MMVP	MV6	376	----	----	24	1200	620	160	5
9% MMVP	MV9	364	----	----	36	1200	620	160	5
12% MMVP	MV12	352	----	----	48	1200	620	160	5

**Table 6:** Compressive and splitting tensile strength results (Mean ± SD) and coefficient of variation (CV%) for all concrete mixtures.

Mix Code	Compressive Strength (7 Days)		Compressive Strength (28 Days)		Compressive Strength (90 Days)		Splitting Tensile Strength (28 Days)	
	Mean ± SD (MPa)	CV (%)	Mean ± SD (MPa)	CV (%)	Mean ± SD (MPa)	CV (%)	Mean ± SD (MPa)	CV (%)
CO	26.4 ± 1.6	6.1	36.9 ± 3	8.18	40.3 ± 3.7	9.12	3.07 ± 0.28	9.25
GD3	27.7 ± 2.7	9.71	38.4 ± 1.5	3.94	42.7 ± 2.1	4.92	3.28 ± 0.32	9.71
GD6	29.6 ± 1.7	5.89	40.7 ± 2.4	5.87	44.3 ± 2.3	5.16	3.33 ± 0.32	9.48
GD9	31 ± 2.1	6.74	42.1 ± 2.9	6.85	48.2 ± 1.9	3.94	3.52 ± 0.27	7.79
GD12	29.2 ± 2.2	7.65	38 ± 1.9	4.87	42.5 ± 2.5	5.88	3.36 ± 0.21	6.12
AFG3	27.3 ± 2	7.35	38.9 ± 2.3	5.84	44.1 ± 3.7	8.43	3.23 ± 0.26	8.17
AFG6	28.6 ± 1.7	5.82	39.4 ± 2.8	7.04	44.7 ± 3.1	6.99	3.42 ± 0.30	8.88
AFG9	30.3 ± 2.7	9.07	42.8 ± 1.5	3.6	46.3 ± 3.5	7.49	3.46 ± 0.29	8.42
AFG12	28.1 ± 1.2	4.27	39.3 ± 0.9	2.33	44 ± 1.4	3.28	3.21 ± 0.12	3.61
MMV3	29 ± 1.1	3.63	39.5 ± 2.3	5.93	43.7 ± 1.6	3.55	3.40 ± 0.15	4.51
MMV6	29.8 ± 1.7	5.7	42.4 ± 3.5	8.2	46.1 ± 3.7	8.1	3.57 ± 0.26	7.24
MMV9	27.4 ± 1.3	4.79	41.3 ± 1.6	3.9	44.1 ± 1.3	2.9	3.25 ± 0.12	3.57
MMV12	25.8 ± 1	3.7	37.8 ± 2.9	7.57	42.3 ± 4.2	9.87	2.90 ± 0.17	5.9

**Table 7:** EDX results (Weight %).

Mix	Composition wt%											
	C K	O K	Mg K	Al K	Si K	Ca K	Fe K	Ti K	K	S K	Na K	Ca/Si
CO	9.26	52.58	------	1.17	8.89	25.66	1.17	------	------	1.27	------	2.89
GD9	5.99	46.88	------	0.57	18.56	25.48	1.38	------	------	1.14	------	1.37
AFG9	8.32	44.69	------	0.55	19.88	24.45	1.62	------	------	------	0.5	1.23
MMV6	12.01	49.68	0.64	4.14	13.31	15.09	3.42	0.25	1.47	------	------	1.13

## Data Availability

The original contributions presented in this study are included in the article. Further inquiries can be directed to the corresponding author.
